# Is it a Reliable Guide or a Source to be Approached with Caution? GenAI in Parents’ Information Search Processes Regarding Their Children

**DOI:** 10.1007/s00431-026-07146-4

**Published:** 2026-06-15

**Authors:** Mustafa EROL, Ahmet EROL

**Affiliations:** 1 Department of Primary Education Program, Yıldız Technical University, Istanbul, Türkiye; 2 Department of Preschool Education Program, Pamukkale University, Denizli, Türkiye

**Keywords:** Parents, Generative artificial intelligence, Children, AI-assisted parenting

## Abstract

Generative artificial intelligence (GenAI) is increasingly used by parents to get information on child care and learning. This study examines how parents use GenAI in their information-seeking process related to their children’s care and learning. It looks at how this shapes decision-making and how parents emotionally interpret these experiences. Thirty parents of children aged 0–3 participated. Researchers collected data with open-ended qualitative questionnaires and analyzed it through inductive content analysis. Findings showed that parents used generative AI for quick information and guidance during periods of uncertainty, emergencies, or developmental questions. They viewed AI as a helpful resource for health, care, behavior, development, and planning activities. Parents did not always use AI suggestions directly; instead, they evaluated them by considering their experiences, their children’s needs, and professional advice. Emotionally, some felt relief, a sense of security, or a sense of being in control, while others felt insecurity, anxiety, or caution.

*Conclusions*: Parents view generative AI as a helpful, easy-to-access resource for child care and learning. They use it most when needing immediate answers or guidance. At the same time, they treat their information with caution by combining it with their own experiences and expert advice. Generative AI now works as a supporting tool for parents, not as a replacement for human judgment or expert help.

## Introduction

With the widespread adoption of information and communication technologies (ICT), the ways parents access information on childcare, development, and learning have undergone a radical transformation. Today, the vast majority of parents, especially those with young children, have access to the internet and mobile devices [1] This digital transformation has not only facilitated parents’ access to information but has also reshaped the nature of parenting practices. Parenting information, traditionally produced by experts and presented in a one-way manner, is increasingly being replaced by more interactive, user-centric, and personalized digital information environments [2]. One of the most striking aspects of this transformation over the past few years is the rapid integration of generative artificial intelligence (GenAI) technologies into daily life. Language models (LLMs) such as ChatGPT and Gemini not only present existing information but also generate new content based on users’ specific contexts [3]. In this respect, generative AI differs from traditional search engines; rather than presenting information based on keyword matching, it generates meaning through user interaction and reconstructs responses [2].

In an era in which AI is becoming increasingly embedded in everyday life, parenting practices are also being reshaped [4]. Many parents face ongoing challenges in raising children and therefore seek advice, guidance, and support [5]. In parenting contexts, AI-supported tools are increasingly used in areas such as childcare, educational planning, behavior management, and daily life organization [6, 7]. These tools may offer important advantages, including personalized recommendations, support for monitoring developmental progress, and assistance with decision-making [8, 9]. They may also facilitate time management, provide rapid access to information, and reduce cognitive load, particularly in moments of uncertainty [10, 11]. At the same time, the use of AI in parenting involves important limitations and risks. Research suggests that AI-generated information is not always contextually appropriate and may lack cultural sensitivity and developmental specificity [12, 13]. Although GenAI responses are often perceived as accurate and easy to understand, they may insufficiently address developmental context, overlook the emotional and relational dimensions of parenting, and offer limited source transparency [2].

Some studies show that individuals find algorithmic recommendations more reliable than human recommendations [14], while others show that they develop significant resistance to AI, especially in health and high-risk decisions [15]. These conflicting findings demonstrate that the use of AI is shaped not only by technical proficiency but also by multidimensional factors, including trust, perceived risk, contextual appropriateness, and user experience. In the context of parenting, a significant portion of current research focuses on the accuracy, effectiveness, or potential of AI systems as intervention tools [10, 11]. When parents seek information in digital environments, they are not only looking for accurate information but also for reassurance, guidance, and emotional support (Jang et al., 2015; [2]. While GenAI can provide quick and accessible responses to these needs, how parents interpret these responses, the extent to which they trust them, and how they integrate them into their decision-making processes are critical areas of research. In this context, AI may be understood not only as a source of information but also as a conversational tool that can shape how parents interpret situations and regulate uncertainty. Therefore, the present study aims to examine how parents use GenAI in their information-seeking processes regarding their children’s care and learning.

## Method

### Participants

The participants of this exploratory qualitative study were 30 parents of children aged between birth and 36 months who had prior experience using GenAI tools for child-related information-seeking. In this study, GenAI experience was defined as having used at least one conversational AI tool, such as ChatGPT, Gemini, Claude, DeepSeek, or a similar system, to seek information, guidance, interpretation, or practical suggestions concerning a child’s care, development, health, learning, behavior, or daily routines. The focus on parents of children aged 0–36 months was considered appropriate because this period is marked by rapid developmental change and frequent parental information-seeking related to feeding, sleep, health, developmental milestones, early learning, and daily care practices.

Participants were recruited in Türkiye through snowball sampling. The initial study invitation was circulated online via the researchers’ professional and community networks to parents with young children. These parents were also invited to share the invitation with other eligible parents. The invitation clearly stated the inclusion criteria: participants had to be parents of a child aged between birth and 36 months and had to have previously used at least one GenAI tool for child-related purposes. To verify eligibility, the online form included screening questions about parental status, the age of the target child, previous GenAI use, the specific tool or tools used, and examples of child-related queries submitted to AI tools. Responses were excluded if participants did not meet the eligibility criteria, if the reported GenAI use was unrelated to child-related information-seeking, or if the response appeared incomplete, inconsistent, or non-serious. Participants were recruited mainly from urban family networks through online communication channels.

Of the 30 participants, 24 were mothers (80.0%), and 6 were fathers (20.0%). Sixteen of the children were boys (53.3%), and 14 were girls (46.7%). The children’s ages ranged from birth to 36 months. 10 parents (33.3%) had infants aged 0–12 months, 10 parents (33.3%) had children aged 13–24 months, and 10 parents (33.3%) had children aged 25–36 months. Participants reported using different GenAI tools in child-related contexts, including ChatGPT, Gemini, Claude, DeepSeek, and other similar systems. In terms of frequency of use, 14 parents (46.7%) reported using GenAI daily, 7 (23.3%) reported using it several times a week, and 9 (30.0%) reported using it occasionally. The majority of participants were between 28 and 39 years of age (n = 19, 63.3%), while 7 participants (23.3%) were between 40 and 49 years old, and 4 participants (13.3%) were between 20 and 27 years old. In terms of educational background, 18 participants (60.0%) held a university degree, 7 (23.3%) had completed postgraduate education, and 5 (16.7%) had completed secondary education. Regarding occupational background, 12 participants (40.0%) were employed in professional occupations, 8 (26.7%) were public-sector employees, 5 (16.7%) were self-employed, and 5 (16.7%) identified primarily as homemakers. Fifteen participants (50.0%) reported having one child, 11 (36.7%) had two children, and 4 (13.3%) had three or more children. In addition, 17 participants (56.7%) stated that the target child was their first child.

Regarding perceived digital/AI literacy, 8 participants (26.7%) described themselves as highly competent users of digital technologies and AI tools, 16 (53.3%) reported moderate competence, and 6 (20.0%) described their competence as limited. Most participants described their socioeconomic status as middle level (n = 21, 70.0%), while 4 (13.3%) reported low and 5 (16.7%) reported high socioeconomic status. Socioeconomic status was classified in the Turkish context according to household monthly income relative to the national minimum wage. Parents earning approximately the minimum wage were classified as low socioeconomic status, those earning around four times the minimum wage as middle socioeconomic status, and those earning eight times or more than the minimum wage as high socioeconomic status. In terms of perceived health literacy, 7 participants (23.3%) described their ability to access, understand, and evaluate health-related information as high, 16 (53.3%) as moderate, and 7 (23.3%) as limited. Summary information about the participants is presented in Table 1.

**Table 1:** Sociodemographic and GenAI-Use Characteristics of Participants

Characteristic	Category/Description	n	%
Participant role	Mother	24	80.0
	Father	6	20.0
Child gender	Boy	16	53.3
	Girl	14	46.7
Child age	0–12 months	10	33.3
	13–24 months	10	33.3
	25–36 months	10	33.3
Parental age	20–27 years	4	13.3
	28–39 years	19	63.3
	40–49 years	7	23.3
Educational background	Secondary education	5	16.7
	University degree	18	60.0
	Postgraduate education	7	23.3
Occupational background	Professional occupations	12	40.0
	Public-sector employees	8	26.7
	Self-employed	5	16.7
	Homemakers	5	16.7
Number of children	One child	15	50.0
	Two children	11	36.7
	Three or more children	4	13.3
Target the child as the first child	Yes	17	56.7
	No	13	43.3
Socioeconomic status	Low	4	13.3
	Middle	21	70.0
	High	5	16.7
Perceived digital/AI literacy	High	8	26.7
	Moderate	16	53.3
	Limited	6	20.0
Perceived health literacy	High	7	23.3
	Moderate	16	53.3
	Limited	7	23.3
GenAI tools reported	ChatGPT	14	46.7
	Gemini	9	30.0
	Claude	2	6.7
	DeepSeek	4	13.3
	Others	1	3.3
GenAI-use frequency	Daily	14	46.7
	Several times a week	7	23.3
	Occasionally	9	30.0

### Data Collection Process and Ethics

Data were collected between February and April 2026 through a qualitative online survey consisting of open-ended written questions administered via Google Forms. The form included open-ended questions designed to explore parents’ experiences of using generative AI in relation to their children’s care, development, and learning. In the form, participants were also asked to describe whether their GenAI use involved one-time queries, repeated conversations, prompt refinement, comparison across tools, or cross-checking AI-generated responses with professionals or other sources. Although written responses allowed flexibility and reflection, they did not allow for probing or follow-up questioning. Because the data consisted of written responses submitted asynchronously through Google Forms, the researchers were not able to ask probing questions, request clarification, or follow up on brief or ambiguous statements. Therefore, the responses should be interpreted as open-ended written accounts rather than interview-based narratives. Specifically, participants were asked: (1) when and for what purpose they first began using AI in the context of child development, (2) what kinds of questions they typically asked and to provide an example they found particularly helpful, (3) how AI-generated responses influenced their decisions regarding their children, (4) how using AI made them feel during the parenting process, and (5) how they evaluated the contributions and limitations of AI in parenting. The use of Google Forms enabled participants to respond in a flexible and reflective manner, without time pressure, and to share their experiences in writing in their own words. Before responding to the form, participants were informed about the purpose and scope of the study and told that the data would be used solely for scientific research purposes.

Participation was voluntary, and no incentives or coercive procedures were involved. Informed consent was obtained through participants’ completion and submission of the online form. To protect confidentiality, responses were processed anonymously, no identifying personal information was reported, and the data were accessible only to the research team. At the same time, the researchers acknowledge that collecting semi-structured written responses through an online form limited opportunities for probing, clarification, and follow-up questioning. Unlike in-depth interviews, this method may restrict the level of contextual and interactional detail that can be obtained from participants. However, the online written format was intentionally preferred because it enabled parents to respond flexibly, asynchronously, and reflectively in relation to sensitive parenting experiences that often occur in emotionally demanding daily routines. In addition, the written format facilitated participation from parents who might have had limited availability for synchronous interviews due to childcare responsibilities. Therefore, the study should be understood as an initial exploratory qualitative investigation aimed at identifying recurring interpretive patterns rather than producing exhaustive individual life narratives.

### Data Analysis

The data were analyzed using inductive content analysis. This approach was preferred because it enables researchers to derive codes and categories directly from the data without relying on predetermined analytic schemes, thereby allowing patterns of meaning to emerge from participants’ own accounts [17, 18]. In line with this approach, the analysis focused on how parents described the reasons for turning to generative AI, the contexts in which they used it, the ways they evaluated AI-generated information, and the meanings they attached to these experiences. The analysis began with repeated readings of all participant responses in order to achieve immersion in the dataset and gain an overall sense of the content. During this stage, the researchers paid close attention to recurrent expressions, emotionally salient statements, and meaning units relevant to parents’ uses and evaluations of AI. Following this initial familiarization, the data were examined line by line, and meaningful segments were assigned initial codes that reflected the core ideas expressed in participants’ responses.

In the next stage, codes that were conceptually similar were grouped together into broader categories. These categories were then compared and revised through an iterative process to ensure that they accurately reflected the data and were meaningfully distinct from one another. As [18]emphasizes, inductive category development requires continuous refinement, abstraction, and comparison to construct a coherent category system grounded in the material. In this process, overlapping categories were merged, ambiguous distinctions were clarified, and higher-order groupings were developed. In the final stage, the resulting categories were organized into a coherent analytic structure representing the major dimensions of parents’ experiences with generative AI. To enhance the transparency of the analysis, the findings were presented with illustrative quotations drawn directly from participants’ written responses. Following the principles of conventional content analysis, this process made it possible to move beyond isolated statements and to produce a systematic and interpretive account of how parents understand, use, and evaluate generative AI in relation to their children’s care and learning [17].

### Trustworthiness

To enhance the trustworthiness of the study, the analysis was guided by the criteria of credibility, dependability, confirmability, and transferability proposed by [19]. Credibility was strengthened through independent and full-dataset coding by both researchers. Rather than dividing the data between the researchers or coding only a subset of responses, both researchers independently read and coded all written responses provided by the 30 participants. In the first cycle of coding, each researcher conducted line-by-line coding and generated initial codes based on meaningful units in the participants’ accounts. This process resulted in an initial pool of 46 codes, including codes such as rapid information seeking, symptom interpretation, developmental norm-checking, activity planning, emotional reassurance, expert confirmation, selective implementation, and distrust of AI-generated information.

After the first coding cycle, the two researchers compared their independently developed code lists. Most codes were conceptually similar, but some differences emerged in the interpretation and labeling of particular responses. For example, some statements were initially coded by one researcher as health-related information seeking, whereas the other researcher interpreted them as emotional reassurance because the participant emphasized anxiety reduction rather than the health issue itself. Similar differences emerged between developmental monitoring and decision-making support, and between pedagogical guidance and behavior management. These discrepancies were not treated as errors but as opportunities to refine the analytic structure. The researchers returned to the original participant statements, discussed the intended meaning of each response, and reached agreement through repeated comparison with the raw data. Through this process, overlapping codes were merged, ambiguous codes were clarified, and the initial code pool was reduced and organized into a more coherent category system. The final structure consisted of three main categories and nine subcategories.

Dependability was addressed by maintaining a clear and traceable analytic process. The researchers documented each stage of the analysis, including repeated reading of the data, initial coding, comparison of independently generated codes, discussion of coding differences, revision of overlapping codes, development of subcategories, and final organization of the category system. The consistency of the codes and categories was examined through several rounds of review. During these reviews, the researchers checked whether each category was internally coherent, meaningfully distinct from other categories, and adequately supported by participant responses. This iterative process helped ensure that the findings were not based on isolated statements but reflected recurring and stable patterns across the dataset.

Confirmability was strengthened by grounding the interpretations in participants’ own written responses. Throughout the analysis, the researchers sought to remain close to the data and avoid imposing predetermined theoretical assumptions on participants’ accounts. All coding, category development, and interpretive decisions were carried out manually rather than through software-assisted procedures. To make the connection between the raw data and the interpretation visible, each main category and subcategory in the findings section was supported with direct quotations from participants. In addition, the researchers repeatedly revisited the original responses during category development to ensure that the final analytic structure reflected participants’ meanings rather than the researchers’ prior expectations. Transferability was supported through detailed reporting of the study context, participant characteristics, recruitment strategy, eligibility criteria, data collection procedure, and analytic decisions. The participants were described in terms of parental role, child age and gender, educational background, occupation, number of children, socioeconomic status, perceived digital/AI literacy, perceived health literacy, GenAI tool preference, and frequency of GenAI use. In line with the exploratory and qualitative nature of the study, transferability was pursued not through statistical generalization, but through contextual depth, analytic transparency, and descriptive richness.

### Findings

The content analysis revealed that parents’ accounts of using GenAI in relation to their children clustered around three overarching categories: (1) contexts and patterns of GenAI use, (2) decision-making and trust calibration, and (3) emotional meanings and perceived limitations. Table 2 presents an overview of the inductively developed category system, including the main categories, subcategories, representative codes, illustrative quotations, and participant references.

**Table 2:** Summary of the Inductive Category System Regarding Parents’ Use of GenAI

Main Category	Subcategory	Representative Codes	Illustrative Quotations	Participant References
Contexts and Patterns of Parents’ GenAI Use	Health-related uncertainty and urgent care	Urgent or ambiguous situations; illness-related questions; symptom interpretation; information overload; preliminary guidance before professional consultation; rapid reassurance	“I started using it when my child first got sick.” (K12) “The need to get a calm, scientifically based, and quick summary of my child’s current developmental crisis led me to this technology.” (K30) “I’m having AI interpret my ultrasound and test results until my doctor’s appointment.” (K9)	K1, K5, K9, K12, K16, K17, K21, K26, K29, K30
	Developmental norm-seeking and monitoring	Age appropriateness; normality checking; height-weight comparison; monthly developmental expectations; sleep/restlessness/colic interpretation; monitoring developmental change over time	“I usually compare whether their age and height are normal according to standard data.” (K13) “I ask if their behaviors are normal for that month.” (K14) “Seeing that a situation I am experiencing is part of the normal developmental process makes me feel calmer and more in control.” (K29)	K13, K14, K15, K22, K28, K29
	Pedagogical guidance, behavior management, and daily routines	Pedagogical responses to difficult questions; behavior guidance; parenting language; crisis communication; age-appropriate toys; activity generation; routine and meal planning	“I ask myself, ‘How should I behave pedagogically?’” (K4) “The AI gave me a practical, calming roadmap…” (K30) “I ask myself, ‘What kind of quality activity can I do with my child?’” (K11)	K4, K7, K11, K19, K26, K27, K30
Decision-Making and Trust	Direct application and high confidence	Immediate implementation; accepting AI responses as correct; perceived usefulness; confidence in clear and practical advice	“I usually try to implement it, thanks to the responses it gives.” (K1) “I implement the responses it gives.” (K14) “It directly affects it.” (K22)	K1, K14, K22, K23
	Filtering and contextual use	Selective implementation; child-specific evaluation; family dynamics; practical applicability; AI as a thinking partner rather than an authority	“I consider it if it makes sense, I don’t directly apply what I get from it.” (K4) “I apply it if it is an appropriate/applicable answer for my child or our family dynamics.” (K15) “AI helps me see different options and view the situation more broadly.” (K28)	K4, K15, K28
	Positioning in conjunction with expert opinion	Professional confirmation; doctor/teacher/specialist consultation; preparation before expert contact; subordinating AI to human judgment in high-stakes issues	“Even if AI gives very good answers, I would definitely ask a human.” (K11) “It doesn’t affect me much; I ask my doctor and act accordingly.” (K16) “I always prefer to make the final decision together with healthcare professionals.” (K29)	K11, K16, K29
Emotional Meanings and Perceived Limitations	Relief, trust, and reduced mental burden	Reduced anxiety; reassurance; sense of control; mental relief; organized guidance; emotional regulation during uncertainty	“I feel a little more relaxed, my anxiety decreases.” (K1) “I receive reassuring responses, my anxiety decreases.” (K9) “Being able to access a more organized, logical roadmap in seconds makes me feel much safer.” (K30)	K1, K9, K29, K30
	Anxiety, insecurity, and a cautious approach	Mistrust; fear of misuse; anxiety about safety; uncertainty about reliability; cautious engagement	“AI makes me anxious; I don’t think it’s a safe program.” (K21) “More anxiety. Because AI is very easy to misuse.” (K26) “Sometimes trust, sometimes anxiety.” (K6)	K6, K21, K26, K28
	Contextual detachment and inability to replace parental intuition	Generalized responses; limited cultural/contextual sensitivity; lack of empathy; emotional thinness; inability to replace parental intuition or professional expertise	“AI has a very robotic approach emotionally.” (K4) “AI cannot fully replace empathy and relationship-based understanding.” (K28) “Parenting is a very personal thing.” (K14)	K4, K14, K23, K27, K28

### Contexts and Patterns of Parents’ GenAI Use

Parents’ accounts showed that GenAI use was closely embedded in everyday caregiving practices and clustered around recurring situations of uncertainty, interpretation, and practical guidance. This category was therefore organized into three subthemes: health-related uncertainty and urgent care, developmental norm-seeking and monitoring, and pedagogical guidance, behavior management, and daily routines.

#### Health-related uncertainty and urgent care

The most salient context in which parents used GenAI was health-related uncertainty. Parents frequently described turning to AI when their child became ill, displayed an unfamiliar symptom, or when they needed rapid preliminary guidance before contacting a professional. In these accounts, AI was valued not simply because it provided information, but because it helped parents organize uncertainty at moments when they felt vulnerable, confused, or overloaded by fragmented online information. For example, K1 explained that they used AI by asking, *“What should I do when my child gets sick,”* while K12 stated, *“I started using it when my child first got sick.”* Similarly, K17 described using AI *“when I suspect my child has an unusual health problem.”*

Parents’ health-related questions covered a wide range of everyday care concerns, including fever, vomiting, cough, gas pain, umbilical cord care, feeding schedules, vaccination schedules, stool color, test result interpretation, and probiotic use. K16 stated that they asked *“questions about feeding and vomiting,”* while K21 said, *“I only asked a question about the color of stool.”* K26 described a more structured use of AI during illness, explaining, *“When my 27-month-old son was sick, I created a probiotic-supported feeding schedule.”* These examples suggest that parents used AI as an accessible first step for making sense of care-related questions, especially when immediate professional guidance was unavailable. However, parents did not always position AI as a substitute for expert judgment. Rather, several participants framed it as a transitional source of orientation used while waiting for professional consultation. K9 stated, *“I’m having AI interpret my ultrasound and test results until my doctor’s appointment.”* This statement illustrates how AI was used to reduce uncertainty temporarily, not necessarily to make final decisions. Some parents also linked AI use to broader changes in family life. K17 emphasized the weakening of intergenerational caregiving support in contemporary nuclear families:

*“I think everyone feels inadequate in parenting today because of nuclear families. In the past, our parents passed down their experiences. That is not the case in our generation. Our lack of knowledge pushes us to find solutions to problems ourselves. We used to take our children to the doctor constantly for the smallest things, day or night. Now, we ask AI directly. This saves us time and money.”*

#### Developmental norm-seeking and monitoring

A second major pattern involved parents’ efforts to understand whether their child’s development, behavior, bodily growth, sleep, feeding, or emotional responses were age-appropriate. Parents used AI to compare their observations with age-related expectations and to decide whether a behavior should be interpreted as typical, temporary, or potentially concerning. K13 stated, *“I usually compare whether their age and height are normal according to standard data,”* while K14 said, *“I ask if their behaviors are normal for that month.”* K22 similarly reported using AI to *“calculate the height and weight measurements that should be taken each month.”* These statements show that GenAI served as a normative reference point through which parents attempted to locate their child’s development within expected developmental ranges. Importantly, developmental norm-seeking was not merely informational. It was closely tied to reassurance and parental self-regulation. When AI helped parents interpret a behavior as part of a typical developmental process, it also appeared to reduce anxiety. K29 expressed this clearly: *“Seeing that a situation I am experiencing is part of the normal developmental process makes me feel calmer and more in control.”* K15 similarly explained that they were often searching for answers to the *“why”* question and described using AI during a period of sleep refusal to understand whether the situation reflected the child’s temperament or a normal developmental transition.

#### Pedagogical guidance, behavior management, and daily routines

A third pattern involved the use of GenAI for practical guidance in everyday parenting. Unlike health-related uncertainty, these uses were not always crisis-driven. Parents also used AI proactively to plan activities, select toys, organize routines, prepare meals, generate child-friendly explanations, and find developmentally appropriate ways of responding to difficult behaviors or questions. Some parents used AI to support pedagogical communication. K11 described asking AI how to respond when their child asked difficult questions, such as *“what does war mean?”* and *“what does death mean?”* K30 similarly reported using AI to learn *“correct sentence patterns recommended by pedagogues,”* while K26 stated that they used AI when *“selecting age-appropriate Montessori toys.”* K27 described using AI to prepare a weekly meal schedule during the complementary feeding period. Behavior management was another important part of this pattern. Parents described using AI both to interpret children’s challenging behaviors and to regulate their own responses in emotionally charged situations. K4 stated, *“I ask myself, ‘How should I behave pedagogically?’”* K27 described turning to AI after reacting with anger and raising their voice, asking how to repair or respond more appropriately. K30 provided a particularly detailed example of this function:

*“The most helpful example for me was on ‘crisis communication’: When my child had a very intense crying fit in the supermarket, I asked how I should approach him. Instead of getting angry and thinking ‘Why won’t he stop?’, the AI gave me a practical, calming roadmap: ‘He’s having an emotional outburst right now; make him feel safe and just wait with him until he calms down.’ This concrete advice, which helped me manage my stress at that moment and connect with my child rather than conflict, really changed my parenting approach.”*

Parents also used AI to enrich play and daily interaction. K11 stated, *“I ask myself, ‘What kind of quality activity can I do with my child?’ and I write down their age, gender, and what I want.”* K19 asked about *“what activities are suitable for a 2-year-old,”* while K30 described AI’s ability to *“generate games or stories in seconds”* based on the child’s interests as a *“lifesaver”* in parenting.

### Decision-Making and Trust

This category addresses how parents positioned AI-generated information in relation to their actual decisions about their children. Rather than a single pattern of acceptance or rejection, the data revealed a continuum of trust calibration ranging from direct application to filtered evaluation and expert-anchored use. Trust in AI was neither uniform nor fixed; it was context-dependent and negotiated according to perceived stakes, child-specific fit, and the availability of human expertise.

#### Direct Application and High Confidence

A smaller but notable group of parents described using AI-generated responses quite directly in their decision-making, regarding the information as sufficiently trustworthy or practical to implement without substantial additional evaluation. K1 stated, "I usually try to implement it, thanks to the responses it gives." K14 similarly said, "*I implement the responses it gives*," and K22 added, "*It directly affects it*." K23’s comment, "*It generally influences my decision-making by making me accept it as correct*," also supports this pattern. For this group, trust appeared to be built primarily on speed, clarity, and the perceived usefulness of the response; AI was treated as cognitively efficient and readily applicable, especially when parents found the answer comprehensible and actionable.

#### Filtering and Contextual Use

More commonly, parents described a selective and interpretive stance toward AI-generated suggestions. Rather than implementing responses as given, they filtered them through their knowledge of their child, family dynamics, practical constraints, and intuition. K4 stated, "*I consider it if it makes sense, I don’t directly apply what I get from it,"* while K15 explained, "*I apply it if it is an appropriate/applicable answer for my child or our family dynamics*." In this pattern, AI functioned as a thinking partner and perspective-expanding resource rather than a decision-making authority. K28 expressed this clearly:

*Rather than directly determining my decisions, the AI responses mostly enrich my thought process. When I face a developmental situation or struggle to understand a behavior, AI helps me see different options and view the situation more broadly. I use AI as a preliminary assessment, a tool for seeing alternatives, and a way to question myself.*

#### Positioning in Conjunction with Expert Opinion

A third pattern involved placing AI explicitly alongside, but beneath, expert or professional judgment. Parents in this group tended to see AI as useful for preparation, clarification, or emotional management while waiting to consult a doctor, teacher, or specialist, but they did not regard it as a final authority. K11 emphasized that even if AI gives very good answers, they would "*definitely ask a human*." K16 said, "*It doesn’t affect me much; I ask my doctor and act accordingly*," while K29 stated, "*I always prefer to make the final decision together with healthcare professionals*." These accounts suggest that trust in AI was calibrated according to the perceived seriousness of the issue and its consequences. In lower-stakes or exploratory situations, AI could function more autonomously; in higher-stakes decisions, parents re-anchored their judgment in human expertise.

### Emotional Meanings and Perceived Limitations

The final category addresses how parents emotionally experienced the use of AI and how they evaluated its boundaries. The analysis brought together emotional meanings and perceived limitations because participants consistently described the benefits and risks of AI in a tightly interwoven manner. Three analytical codes were identified: relief, trust, and reduced mental burden; anxiety, insecurity, and cautious approach; and contextual detachment and the inability to replace parental intuition.

#### Relief, Trust, and Reduced Mental Burden

Many participants described AI as emotionally relieving, particularly in moments of uncertainty. They reported that using AI reduced anxiety, provided reassurance, and created a sense of control. K1 expressed this as, "*I feel a little more relaxed, my anxiety decreases*," while K9 similarly stated, "*I receive reassuring responses, my anxiety decreases*." This affective function became especially visible when AI helped parents interpret a situation as developmentally normal or rendered chaotic information more coherent. Importantly, this feeling of relaxation was linked not only to acquiring information but also to seeing the situation as part of an expected developmental process (K29), suggesting that AI served both as an informational tool and as an emotional regulation mechanism. K30 describes this experience comprehensively:

*The use of AI creates the most “mental relief” and “a sense of control” in my parenting process. While I used to get lost and anxious amid conflicting information from dozens of sources when faced with a problem, now being able to access a more organized, logical roadmap in seconds makes me feel much safer. Especially when no one is around, being able to get an objective and calm answer to the question “Am I doing something wrong?” in my head lightens the heavy burden of parenting.*

#### Anxiety, Insecurity, and Cautious Approach

The experience was not uniformly positive. Some parents questioned the reliability of AI and described it explicitly as a source of anxiety. K21 said, "*AI makes me anxious; I don’t think it’s a safe program*." K26 stated, "*More anxiety. Because AI is very easy to misuse*." K6’s statement, "*Sometimes trust, sometimes anxiety*," clearly reflects this emotional duality. These responses indicate that parents’ emotional relationship with AI was not uniformly positive; its reassuring function was accompanied by an awareness that information may be incomplete, inappropriate, or misleading. K28 summarized this dual experience as "*a tool that provides reassurance when used in a controlled manner, but also reminds us that caution is needed*." This awareness produced a cautious mode of engagement in which usefulness and suspicion coexisted.

#### Contextual Detachment and Inability to Replace Parental Intuition

The most frequently cited limitation concerned AI’s lack of contextual sensitivity. Parents repeatedly noted that AI-generated responses were often general, standardized, and emotionally thin. K4 stated that AI has "*a very robotic approach emotionally*," which is why they could not apply its responses directly without filtering. K27 addressed this limitation in detail:

*Since it is not a real expert, its ability to generalize and interpretations that can be considered detached from culture, and most importantly, its inability to guide towards the principle of child-centeredness, can be considered a limitation. Using only AI to make decisions or implement practices regarding children can lead to incorrect results and be dangerous.*

K28 similarly stated that "*in emotionally charged parenting processes, AI cannot fully replace empathy and relationship-based understanding*." Participant narratives showed strong consensus that AI cannot replace parental intuition, individual observation, or expert knowledge. K23 summarized this view by saying, "*I think what matters, maternal instinct and emotions*," while K14 implied that AI’s general outputs cannot align with the uniqueness of individual children, stating, "*Parenting is a very personal thing*." These accounts suggest that parents did not dismiss AI altogether; rather, they indicated that it had a limited and conditional role.

## Discussion

The present study examined how parents use generative AI in information-seeking processes related to their children’s care and learning, how they position AI-generated information within decision-making, and how they emotionally interpret this experience. The findings suggest that parents do not use GenAI merely as a conventional source of information. Rather, they turn to it as an interpretive and practical support tool, particularly when they face uncertainty, developmental questions, or the need for immediate guidance. GenAI use was most visible in three interconnected contexts: health-related concerns, developmental norm-seeking, and everyday pedagogical support. In these contexts, parents used AI to make sense of symptoms, compare their child’s behavior or development with age-related expectations, generate activity and routine ideas, and prepare responses to challenging child-related situations.

This pattern shows that GenAI occupies a provisional space in parenting: it helps parents organize concerns before professional consultation, supports reflection on everyday caregiving decisions, and offers practical suggestions for managing routine family life. Consistent with previous research, parents’ digital information-seeking was not limited to obtaining factual answers; it also involved reassurance, orientation, and emotional support (Jang et al., 2015; [2]. At the same time, the findings extend earlier work by showing that GenAI is not used only in moments of crisis. Parents also drew on it proactively, for example, when planning age-appropriate activities, selecting materials, structuring routines, or formulating developmentally sensitive responses to children’s questions. Health and care management emerged as the most prominent area of use. Parents frequently used AI to interpret symptoms, organize care-related questions, and obtain preliminary guidance while waiting for professional advice or when immediate support was unavailable. This finding is consistent with studies suggesting that AI can function as a first-step guidance tool without replacing professional expertise [16, 20]. The contribution of the present study lies in showing how this support operates in the temporal gap between parental concern and expert consultation, while also extending to developmental monitoring, behavior guidance, parenting language, play, activities, and daily routines.

The findings related to behavior management and parenting language are equally noteworthy. Parents did not use AI only to interpret their children’s behavior; they also used it to regulate their own responses, especially in emotionally intense moments. In this respect, AI seems to serve not only as a source of child-focused guidance but also as a scaffold for parental self-regulation. Some participants described using AI after moments of anger, confusion, or escalation, seeking a more developmentally sensitive or pedagogically appropriate way of responding. These accounts suggest that AI may, in some cases, create a brief reflective space in which parents reconsider their immediate reactions. Although this interpretation should be made cautiously, it resonates with research showing that AI-based conversational tools may support parental learning, reflection, and skill development [11, 21].

Rather than revealing a simple divide between trust and distrust, the findings point to a continuum ranging from direct application to filtered and contextual use, to expert-anchored positioning. This is a particularly important result because it shows that trust in AI is not a fixed attitude but a negotiated process. Some parents were willing to apply AI suggestions rather directly, especially when the advice appeared clear, practical, and low risk. More commonly, however, parents filtered the information through their own experience, their child’s characteristics, and the realities of family life. In higher-stakes issues, especially health-related decisions, AI was often explicitly subordinated to professional judgment. This pattern is consistent with research demonstrating that people may appreciate algorithmic advice under some conditions while resisting it in more sensitive domains [14, 15]. This trust calibration perspective offers a more nuanced interpretation than simply stating that parents either trust or distrust AI. The findings suggest that parents actively evaluate the legitimacy of AI-generated information according to issue severity, perceived risk, contextual fit, and the availability of human expertise. This interpretation is in line with [2, 12], who note both the usefulness and the limitations of generative AI in family and early childhood contexts.

The final category, emotional meanings and perceived limitations, further reinforces this negotiated character. The findings reveal a clearly ambivalent emotional pattern. On the one hand, many parents experienced AI as relieving, reassuring, and mentally lightening. Especially when faced with uncertainty, AI seems to reduce cognitive overload by translating dispersed or conflicting information into a more organized and accessible response. This finding is compatible with prior literature suggesting that digital parenting supports may reduce stress and mental burden [10, 22]. Some parents worried that AI could be misleading, misused, or overly general. It suggests that the emotional role of AI in parenting is not simply supportive, but ambivalent: the same technology that reduces anxiety may also generate new uncertainty once its limitations are recognized. Such findings indicate that parents’ relationships with AI are not emotionally settled. Rather, they remain provisional and reflexive, marked by simultaneous appreciation and doubt. This aligns with broader discussions in the AI literature emphasizing that technological usefulness does not eliminate concerns about trustworthiness, context sensitivity, and human judgment [23].

Perhaps the most critical boundary identified by parents concerns contextual detachment. Participants repeatedly emphasized that AI-generated responses may be informative, but are often too general, emotionally thin, culturally decontextualized, or insufficiently attuned to the uniqueness of a particular child. This finding is highly consistent with previous critiques of AI use in child and family contexts, where concerns have been raised about limited developmental specificity, inadequate cultural sensitivity, and the absence of relational understanding [2, 12, 13].

### Limitations and Future Research

This study has several limitations that should be considered when interpreting the findings. The participant group was composed predominantly of mothers, which means that the findings may reflect parenting experiences primarily from a maternal perspective. Another limitation concerns the sociodemographic profile of the participants. Most participants had a university or postgraduate educational background and identified themselves as belonging to a middle socioeconomic status group. In addition, many participants described themselves as moderately or highly competent in digital and AI-related technologies. These characteristics suggest that the findings may primarily reflect the experiences of parents with relatively greater access to digital technologies, information resources, and AI-supported environments. Parents with lower levels of education, limited digital literacy, or restricted access to technological resources may engage with generative AI differently or may experience additional barriers, concerns. A more balanced inclusion of mothers and fathers in future research would make it possible to examine whether parents differ in how they use, interpret, and evaluate generative AI in relation to their children.

Another limitation concerns the nature of the data collection process. The study relied entirely on self-reported written responses gathered through Google Forms. For this reason, participants’ accounts may reflect perceptions, retrospective interpretations, and selective recall rather than their actual real-time practices. In addition, although the written format offered flexibility and allowed participants to respond reflectively, it did not allow for probing, clarification, or follow-up questions. This limitation is particularly important because GenAI use may involve complex practices such as iterative prompting, reformulating questions, comparing multiple responses, or selectively applying advice. Such practices are difficult to capture fully through written survey responses alone. Therefore, although the present data provide useful initial insights, they may not reflect the full depth, sequence, or interactional nature of parents’ real-time engagement with GenAI tools. In addition, some participant responses were relatively brief and did not provide extensive narrative elaboration. Although these responses were analytically useful for identifying recurring patterns, they should not be interpreted as rich interview narratives. Future studies using in-depth interviews, follow-up questions, or think-aloud protocols could provide a more detailed understanding of how parents formulate prompts, interpret AI-generated responses, and integrate them into real-time parenting decisions.

The age range of the children represented in the study also narrows the scope of the findings. Another limitation is that the study did not systematically assess whether the participating children had diagnosed developmental, medical, or educational difficulties. This is important because parents of children with known developmental or health-related needs may use GenAI differently, particularly for developmental monitoring, symptom interpretation, and care-related decision-making. Future studies should collect this information explicitly and examine how children’s developmental, medical, or educational status may shape parents’ use, trust, and interpretation of GenAI-generated guidance. Because most participants were parents of children in infancy and early childhood, the results are especially relevant to these developmental periods and may not fully capture how generative AI is used in relation to older children. Future research should therefore explore whether the purposes, meanings, and consequences of AI use vary across developmental stages. Finally, the present study focused on parents’ reported uses and interpretations of generative AI rather than on the direct effects of AI-assisted parenting on child outcomes. For this reason, future work should move beyond descriptive accounts of parental use and investigate how AI-mediated guidance may shape parental decision-making, parent–child interactions, and children’s cognitive, emotional, and social development. Longitudinal, mixed-method, and experimental studies would be especially valuable in clarifying these processes. Further research is also needed to examine the ethical, cultural, and socioeconomic dimensions of AI use in parenting, particularly how access, digital literacy, and contextual resources shape parents’ engagement with these technologies. [24]

## Conclusion

This study shows that generative AI has become a meaningful part of parents’ information-seeking practices regarding their children’s care, development, and learning. Parents used GenAI mainly in situations marked by uncertainty, developmental concern, or the need for practical guidance. However, their engagement with AI was not characterized by passive acceptance or uncritical dependence. Rather, parents evaluated AI-generated responses in relation to their own experiences, their child’s individual characteristics, family context, and professional advice. The findings suggest that GenAI functions as a provisional support tool in parenting. It can help parents organize fragmented information, reduce uncertainty, generate practical ideas, and reflect on possible responses to everyday caregiving situations. Parents recognized that AI cannot replace professional expertise, emotional attunement, parental intuition, or the relational and context-sensitive nature of caregiving. Therefore, the value of GenAI in parenting lies not in autonomous decision-making, but in its potential to support parental reflection and informed judgment. Given its exploratory qualitative design, this study should be understood as an initial account of how parents make sense of GenAI in everyday caregiving. Rather than positioning GenAI as a substitute for professional expertise, practitioners and educators may help parents develop critical and reflective ways of using AI-generated guidance in everyday caregiving and decision-making processes.

## Data Availability

The datasets generated and/or analyzed during the current study are available from the corresponding author on reasonable request, in accordance with ethical and privacy considerations.
